# Prognostic value of systemic inflammatory markers for oral cancer patients based on the 8th edition of AJCC staging system

**DOI:** 10.1038/s41598-020-68991-3

**Published:** 2020-07-21

**Authors:** Sanghoon Lee, Dong Wook Kim, Sunmo Kwon, Hyung Jun Kim, In-Ho Cha, Woong Nam

**Affiliations:** 1 Oral Oncology Clinic, Research Institute and Hospital, National Cancer Center, 323, Ilsan-ro, Ilsandong-gu, Goyang-si, Gyeonggi-do 10408 Republic of Korea; 2 Department of Oral and Maxillofacial Surgery, Yonsei University College of Dentistry, 50-1, Yonsei-ro, Seodaemoon-gu, Seoul, 03722 Republic of Korea

**Keywords:** Oral cancer, Surgical oncology, Risk factors

## Abstract

It has been recognized that systemic inflammatory markers (SIMs) are associated with patient survival in various types of cancer. This study aimed to determine the optimal cut-off values, and to evaluate the prognostic performance of SIMs for oral squamous cell carcinoma (OSCC) within the framework of the American Joint Committee of Cancer (AJCC) cancer staging manual, 8th edition. Records were collected for a total 291 patients who had had a peripheral blood test within 1 week prior to surgery and had undergone the surgical resection of OSCC in a single institution between 2005 and 2018. The cut-off values of SIMs were obtained, and the survival analyses for overall survival (OS) and disease-free survival (DFS) were performed. Multivariate analyses incorporating other clinicopathologic factors were performed to verify the independent risk factors for survival. The cut-off values of neutrophil-to-lymphocyte ratio (NLR) and platelet-to-lymphocyte ratio (PLR) were 2.23, 135.14 for OS and 2.16, 131.07 for DFS, respectively, demonstrating a significant association for OS and DFS in OSCC. AJCC pathologic regional lymph node category (pN) (*P* < 0.001), perineural invasion (PNI) (*P* < 0.001) and NLR (*P* < 0.001) were independent predictors for OS. Meanwhile, for DFS, AJCC pN (*P* = 0.018) and NLR (*P* = 0.015) were shown to be independent predictors. Before the curative surgery, NLR and PLR could be auxiliary parameters for OS and DFS in OSCC. And based on the 8th edition of AJCC staging system, elevated NLR will be a potential indicator of the worse OS or DFS along with pN or PNI in OSCC.

## Introduction

The ‘primary tumor, regional lymph node and distant metastasis’ (TNM) staging system has been generally accepted as a worldwide classification tool for management of oral squamous cell carcinoma (OSCC)^1–4^. The product of constant revisions to improve the prognostic stratification, the 8th edition of the American Joint Committee of Cancer (AJCC) cancer staging manual was recently published^4^. However, this staging framework has focused on the clinical and pathological characteristics of tumor rather than host factors and most of the pathological data can be identified postoperatively^5^. Accordingly, any prognostic factor that can be obtained before surgery might be valuable in establishing a treatment plan for OSCC.

Among host factors in cancer initiation and progression which have been investigated, inflammatory condition is known as one of the hallmarks^3,6^. Research on interactions between tumor development and systemic inflammation indicates that chronic inflammation can stimulate carcinogenesis, the degree of systemic inflammation correlating with oncologic outcomes^7–9^. Peripheral blood sampling is a simple and useful modality for measuring the systemic inflammation of patients in clinical situations^10,11^. Based on the peripheral blood differential counts, several combined indices have been suggested as prognostic markers. Systemic inflammatory markers (SIMs) including neutrophil-to-lymphocyte ratio (NLR), lymphocyte-to-monocyte ratio (LMR), platelet-to-lymphocyte ratio (PLR) and albumin are significantly associated with the survival of OSCC patients^12–15^. However, there are discrepancies in the prognostic impact and cut-off values among those SIMs for OSCC.

The aim of the present study was to verify the prognostic significance of preoperative SIMs, including NLR, LMR, PLR and albumin for the management of OSCC, within the framework of the AJCC cancer staging manual, 8th edition, as well as to determine the optimal cut-off value of SIMs for OSCC patients who are undergoing definitive surgery.

## Material and methods

### Patient demographics and clinical data

This study retrospectively enrolled adult patients (18 years or older) who had been newly diagnosed with OSCC and undergone curative surgery without neoadjuvant therapy at Yonsei University Dental Hospital from November 1, 2005, through August 31, 2018. Only patients who had had a peripheral blood test within 1 week prior to surgery were included in the study. Exclusion criteria included the following conditions: patients who had other concomitant primary cancer, distant metastatic cancer, perioperative mortality, a history of previous head and neck cancer, previous radiotherapy and/or chemotherapy, hematological disorders, infection, inflammatory conditions, autoimmune disease, administration of steroids or for whom preoperative laboratory data within 1 week before surgery was lacking. Initially, 496 patients were identified. After excluding 205 patients for insufficient data or meeting the exclusion criteria, a total of 291 patients were evaluated. Demographic, laboratory, and clinical data were analyzed. Information was collected on any comorbidity at the time of OSCC diagnosis and the Charlson comorbidity index (CCI) was calculated. A high comorbidity score was defined as a CCI of ≥ 3. Disease staging was based on the 8 h edition of the AJCC cancer staging manual (2018). LMR was calculated by dividing the absolute lymphocyte count by the absolute monocyte count. NLR was calculated by dividing the absolute neutrophil count by the absolute lymphocyte count. PLR was calculated by dividing the absolute platelet count by the absolute lymphocyte count.

The Ethics Review Board of Yonsei University Dental Hospital Institutional Review Board approved the study (IRB No. 2-2018-0047) and accepted that informed consent was not required as the study had a non-interventional retrospective design and all data were analyzed anonymously. All procedures of the study involving human participants were in accordance with the Declaration of Helsinki (1964) and its later amendments or comparable ethical standards. All authors had access to the study data and reviewed and approved this study.

### Statistical analysis

A receiver operating characteristic (ROC) curve analysis was done in order to obtain the cut-off values of LMR, NLR, PLR and albumin for overall survival (OS) and disease-free survival (DFS). The values with maximal sensitivity and specificity were selected for analysis. OS was calculated from the date of surgery to death from any cause. DFS was calculated from the date of surgery to the date of recurrence, or death from any cause. The Kaplan–Meier curve was used to analyze patients’ survival and the survival outcomes were assessed with a log-rank test. If the patient survived without an event, survival was censored at the latest date of follow-up when no event was confirmed. Univariate and multivariate analyses were done to identify independent risk factors for survival using Cox proportional hazards regression models. All analyses were performed using IBM SPSS Statistics for Windows, version 23.0 (IBM Corp., Armonk, NY, USA). A *P* value of < 0.05 was considered to be statistically significant.

## Results

### Baseline characteristics

The demographic and clinicopathological characteristics of 291 patients are presented in Table 1. The mean follow-up period for surviving patients was 41 months (range 3–144 months). There were 183 men and 108 women, median age at diagnosis being 63 years (range 24–91). The most common primary site was mandibular gingiva, followed by tongue and buccal cheek mucosa. Patients were divided according to the 8th edition of the AJCC TNM staging manual: stage I (n = 67, 23.0%); stage II, (n = 63, 21.6%) stage III (n = 35, 12.0%); stage IVA (n = 89, 30.6%); stage IVB (n = 37, 12.7%). A ma jority of the enrolled patients had clinically N0 disease (207/291, 71.1%), and a relatively better histologic grade ranging from moderately to well-differentiated disease (199/291, 68.4%). Less than half of the patients received adjuvant treatment after the surgery (120/291, 41.2%) and approximately three-quarters of the patients survived (50/291, 17.2%).

**Table 1:** Clinicopathologic characteristics of patients with oral squamous cell carcinoma.

Parameter	Number (%)
Total	291
**Age, years**	
Median (range)	63 (24–91)
< 63 years	142 (48.8)
≥ 63 years	149 (51.2)
**Sex**	
Male	108 (37.1)
Female	183 (62.9)
**Smoking history**	
Yes	183 (62.9)
No	108 (37.1)
**Alcohol history**	
Yes	161 (54.9)
No	132 (45.1)
**CCI**	
0–2 points	283 (97.3)
≥ 3 points	8 (2.7)
**Location of primary tumor**	
Tongue	77 (26.5)
FOM	22 (7.6)
RMT	36 (12.4)
Mandibular gingiva	85 (29.2)
Lip	7 (2.4)
Buccal cheek mucosa	42 (14.4)
Maxilla	22 (7.6)
**AJCC pT categories**	
pT1	78 (26.8)
pT2	85 (29.2)
pT3	35 (12.0)
pT4a	91 (31.3)
pT4b	2 (0.7)
**AJCC pN categories**	
pN0	199 (68.4)
pN1	21 (7.2)
pN2	35 (12.0)
pN3	36 (12.4)
**AJCC staging**	
I	67 (23.0)
II	63 (21.6)
III	35 (12.0)
IVA	89 (30.6)
IVB	37 (12.7)
**Adverse pathologic features**	
Lymphovascular permeation	39 (13.4)
Perineural invasion	33 (11.3)
Extranodal extension	40 (13.7)
NA	1 (0.3)
**Resection margin status**	
Positive	28 (9.6)
Close ($\le$ 5 mm)	93 (32.0)
Negative (> 5 mm)	169 (58.1)
NA	1 (0.3)
**Adjuvant therapy**	
RTx alone	53 (18.2)
CTx alone	2 (0.7)
CCRT	65 (22.3)
None	171 (58.8)
**Relapse**	
Loco-regional relapse	66 (22.7)
Distant relapse	1 (0.3)
None	224 (77.0)
**Survival status**	
Alive	241 (82.8)
Dead	50 (17.2)

*CCI* Charlson comorbidity index, *FOM* floor of mouth, *RMT* retromolar trigone, *AJCC* American Joint Committee on Cancer, *pT* pathologic primary tumor categories, *pN* pathologic regional lymph node categories, *NA* not applicable, *RTx* radiotherapy, *CTx* chemotherapy, *CCRT* concurrent chemoradiotherapy.

### Cut-off values of SIMs

Differential white blood cell count, calculated ratios and albumin are shown in Table 2. The mean NLR, LMR, PLR and albumin were 2.61, 5.01, 141.26 and 4.34 and the medians were 2.04 (range (0.50–32.36), 4.58 (0.67–14.63), 127.27 (45.95–655.56) and 4.40 (2.40–5.60), respectively. According to the ROC curve analysis, the cut-off values of SIMs were separately determined for OS and DFS. The cut-off values of NLR, LMR, PLR and albumin were 2.23, 4.65, 135.14 and 4.35 for OS and 2.16, 4.45, 131.07 and 4.35 for DFS, respectively ***(Supplementary Tables S1, S2; Figures S1, S2).

**Table 2:** Inflammatory markers, calculated ratios and cut-off values in patients with oral squamous cell carcinoma.

Parameter	Mean ± SD	Median $(range)$	Cut-off value	
			OS	DFS
**Differential white blood cell count**				
Neutrophil count, 10^9^/L	4.38 ± 2.10	3.92 (1.52–17.15)	NA	NA
Lymphocyte count, 10^9^/L	1.98 ± 0.67	1.90 (0.32–3.95)	NA	NA
Monocyte count, 10^9^/L	0.43 ± 0.17	0.39 (0.10–1.08)	NA	NA
Platelet count, 10^9^/L	248.04 ± 66.54	247.00 (48.0–634.0)	NA	NA
**Calculated ratios**				
NLR	2.61 ± 2.52	2.04 (0.50–32.36)	2.23	2.16
LMR	5.01 ± 2.09	4.58 (0.67–14.63)	4.65	4.45
PLR	141.26 ± 72.24	127.27 (45.95–655.56)	135.14	131.07
Albumin	4.34 ± 0.37	4.40 (2.40–5.60)	4.35	4.35

*OS* overall survival, *DFS* disease-free survival, *NA* not applicable, *NLR* neutrophil–lymphocyte ratio, *LMR* lymphocyte-monocyte ratio, *PLR* platelet-lymphocyte ratio.

### Survival analysis according to the SIMs

**Figure 1:**
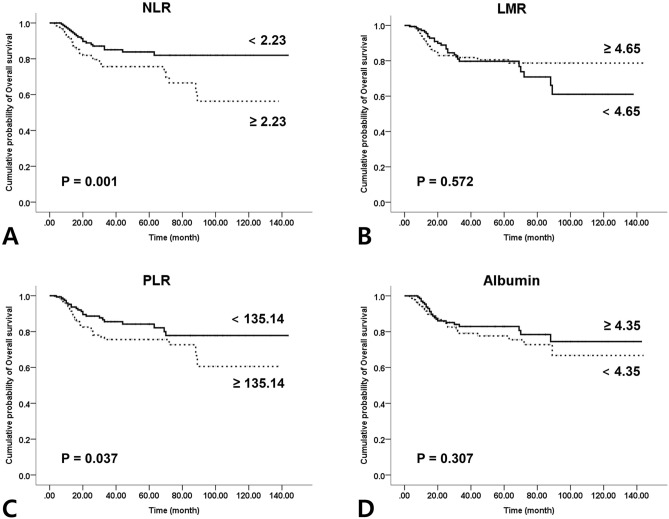
Kaplan–Meier curves for overall survival according to the preoperative systemic inflammatory markers: (**A**) neutrophil–lymphocyte ratio (P = 0.001); (**B**) lymphocyte–monocyte ratio (P = 0.572); (**C**) platelet–lymphocyte ratio (P = 0.037); (**D**) Albumin (P = 0.307).

**Figure 2:**
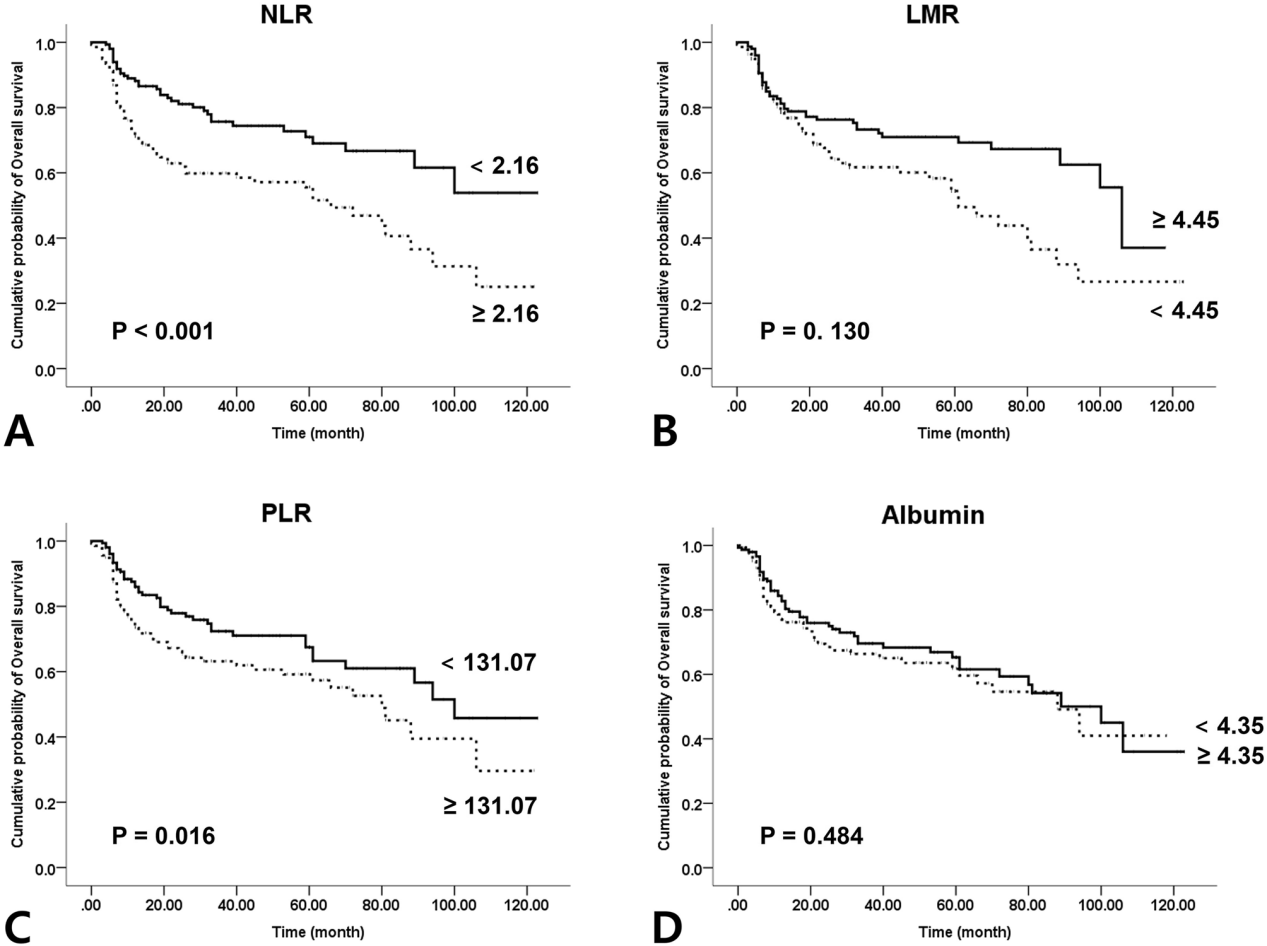
Kaplan–Meier curves for disease-free survival according to the preoperative systemic inflammatory markers: (**A**) neutrophil–lymphocyte ratio (P $<$ 0.001); (**B**) lymphocyte–monocyte ratio (P = 0.130); (C) platelet–lymphocyte ratio (P = 0.016); (**D**) Albumin (P = 0.484).

The OSCC patients were divided into two groups according to the cut-off values for Kaplan–Meier analysis. NLR showed statistically significant association with both OS and DFS (*P* = 0.001 and *P* < 0.001). PLR also showed statistically significant association with both OS and DFS in OSCC patients (*P* = 0.037 and *P* = 0.016). A trend towards better survival was observed for patients with higher LMR and albumin, but the results lacked statistical significance for both OS and DFS (*P* = 0.572, 0.307 and *P* = 0. 130, 0.484) (Figs. 1, 2).

### Cox proportional hazards regression model

The Cox proportional hazards regression model revealed that AJCC pathologic regional lymph node category (pN) [converted into a binomial variable of N2, 3 vs. 0, 1; hazard ratio (HR) 2.29, 95% confidence interval (CI) 1.27–4.10, *P* < 0.001], perineural invasion (PNI) (HR 2.29, 95% CI 1.27–4.10, P < 0.001) and NLR (≥ 2.23 vs. < 2.23; HR 2.29, 95% CI 1.27–4.10, *P* < 0.001) were independent predictors for OS (Table 3). For DFS, AJCC pN (N2, 3 vs. 0, 1; HR 1.70, 95% CI 1.10–2.65, *P* = 0.018) and NLR (≥ 2.16 vs. < 2.16; HR 1.82, 95% CI 1.12–2.94, *P* = 0.015) were shown to be independent predictors (Table 4).

**Table 3:** Cox proportional hazard regression model for overall survival.

Parameter	Univariate analysis		Multivariate analysis	
	HR (95% CI)	*P* value	HR (95% CI)	*P* value
Age (≥ 63)	1.31 (0.75–2.29)	0.341		
Smoking history	0.85 (0.47–1.55)	0.601		
Alcohol history	1.00 (0.57–1.76)	0.989		
CCI (≥ 3 points)	0.58 (0.00–134.19)	0.453		
AJCC pT (T3, T4a and T4b)	1.74 (0.10–3.05)	0.051		
AJCC pN (N2, N3)	2.35 (1.34–4.11)	< 0.001*	2.29 (1.27–4.10)	0.006*
Perineural invasion	2.89 (1.47–5.66)	0.002*	2.17 (1.09–4.33)	0.028*
Lymphovascular permeation	1.78 (0.89–3.57)	0.102		
Resection margin status (close or involved)	0.88 (0.49–1.58)	0.676		
NLR (≥ 2.23)	2.01 (1.15–3.53)	0.015*	1.78 (1.01–3.14)	0.045*
LMR (< 4.65)	0.88 (0.50–1.53)	0.638		
PLR (≥ 135.14)	1.67 (0.96–2.92)	0.071		
Albumin (< 4.35)	1.31 (0.75–2.29)	0.340		

*HR* hazard ratio, *CI* confidence interval, *CCI* Charlson comorbidity index, *AJCC* American Joint Committee on Cancer, *pT* pathologic primary tumor categories, *pN* pathologic regional lymph node categories, *NLR* neutrophil–lymphocyte ratio, *LMR* lymphocyte-monocyte ratio, *PLR* platelet-lymphocyte ratio.

* Statistically significant.

**Table 4:** Cox proportional hazard regression model for disease-free survival.

Parameter	Univariate analysis		Multivariate analysis	
	HR (95% CI)	*P* value	HR (95% CI)	*P* value
Age (≥ 63)	1.24 (0.83–1.84)	0.287		
Smoking history	0.72 (0.47–1.11)	0.137		
Alcohol history	0.99 (0.66–1.47)	0.941		
CCI (≥ 3 points)	0.89 (0.22–3.63)	0.872		
AJCC pT (T3, T4a and T4b)	1.41 (0.95–2.09)	0.089		
AJCC pN (N2, N3)	2.08 (1.36–3.17)	0.001*	1.70 (1.10–2.65)	0.018*
Perineural invasion	2.015 (1.190–3.412)	0.009*		
Lymphovascular permeation	1.40 (0.83–2.37)	0.204		
Resection margin status (close or involved)	1.37 (0.92–2.04)	0.122		
NLR (≥ 2.16)	2.10 (1.39–3.15)	< 0.001*	1.82 (1.12–2.94)	0.015*
LMR (< 4.45)	1.65 (1.11–2.47)	0.014*		
PLR (≥ 131.07)	1.55 (1.04–2.30)	0.032*		
Albumin (< 4.35)	1.17 (0.79–1.73)	0.439		

*HR* hazard ratio, *CI* confidence interval, *CCI* Charlson comorbidity index, *AJCC* American Joint Committee on Cancer, *pT* pathologic primary tumor categories, *pN* pathologic regional lymph node categories, *NLR* neutrophil–lymphocyte ratio, *LMR* lymphocyte-monocyte ratio, *PLR* platelet-lymphocyte ratio.

*Statistically significant.

## Discussion

In the present study, we investigated SIMs as prognostic factors that can simply be analyzed before surgery on OSCC. Based on the cut-off value and Kaplan–Meier survival analysis, we confirmed that elevated NLR and PLR are negative predictors for OS and DFS. Meanwhile, LMR or albumin did not present any significant correlation with survival. However, there is a discrepancy among the literature regarding the prognostic impact of SIMs. Several researchers have also documented that NLR is significantly associated with OS, DFS or disease specific survival (DSS) of OSCC patients^3,10,14,16,17^. And a recent study proposed a systemic immune-inflammation index (SII) calculated by dividing a multiplication of the absolute neutrophil and platelet count by the absolute lymphocyte count. Diao et al. highlighted the results that a higher SII indicates a poor prognosis for OS and DFS in OSCC patients^18^. Kao et al. presented a nomogram incorporating only NLR and albumin for OS prediction in OSCC patients^5^. Ong et al. demonstrated that LMR and PLR, not NLR, were independent prognostic indicators for OS and DFS in early stage (pT1N0 or pT2N0) tongue cancer^7^. In the study reported by Chen et al., PLR rather than NLR displayed significant associations with OS and DFS of OSCC patients^19^. Further multicenter research with a large population remains to be performed for a worldwide consensus of SIMs.

We also performed multivariate analysis using the Cox proportional hazards regression model with the clinicopathologic parameters that were obtained after surgery, including the depth of invasion and extranodal extension (ENE) according to the AJCC cancer staging manual, 8th edition. pN and NLR were found to be independent prognostic factors for both OS and DFS in OSCC patients and PNI was another significant indicator for OS. These factors are also mentioned in previous literature, but based on the 7th edition of the AJCC cancer staging Manual^16,20^. A staging system proposed by Lee et al., composed of primary tumor category (pT), pN, PNI and NLR, demonstrated better prognostic discrimination compared to the 7th edition of the AJCC staging system for OSCC^16^. Mattavelli et al. also analyzed clinicopathologic and inflammatory factors of which ENE, PNI and NLR were significant prognostic indicators for survival of OSCC patients^21^. To the best of our knowledge, the present study is the first to focus on SIMs in the framework of the 8th edition of AJCC cancer staging Manual. The result of the present study indicated that peripheral blood markers of host inflammation could be a supplementary indicator of survival in the current TNM staging system for OSCC.

NLR has been documented as a valuable predictor and its cut-off value has been examined in various types of cancer: NLR ≥ 3.5 for esophageal squamous cell carcinoma (ESCC)^22^, ≥ 2.7 for intrahepatic cholangiocarcinoma^23^, ≥ 2.5 for DFS in melanoma^24^ and ≥ 2.36 for DFS and DSS in gastric cancer^25^ were associated with poorer survival outcome. With regard to OSCC, the cut-off values of NLR were reported to range from 1.9 to 2.95 for OS and from 1.9 to 2.95 for DSS^3,7,20,26–30^. In the present study, the cut-off values of NLR as 2.23 for OS and 2.16 for DFS. Additionally, PLR and its threshold have also been investigated in diverse cancer types: PLR ≥ 149 for pancreatic cancer^31^, ≥ 138.35 for cervical cancer^32^, ≥ 181.1 and 185.5 for breast cancer^33,34^ and ≥ 150 for ESCC^22^ were associated with poorer survival outcome. As for OSCC, although there is limited insufficient data, the cut-off values of PLR ranged from 124.8 to 138.47 for OS^7,19,35–37^. In this study, we found the cut-off values of PLR to be 135.14 for OS and 131.07 for DFS. Our findings were broadly consistent with those in previous publications. This might be because most of the cut-off values of NLR and PLR for OSCC patients have been predominantly derived from the Asian population; data on the Caucasian population are still lacking.

Recently, reference values of NLR and PLR in the healthy general population have been reported for the Republic of Korea^38^. The mean of NLR and PLR among OSCC patients in this study was generally higher than those of reference mean values from the healthy population. This might be another clue of systemic inflammatory condition associated with carcinogenesis in OSCC patients. Although the mechanism of interaction between systemic inflammation and cancer has not been fully explained, theoretical backgrounds for cancer-related inflammation have gradually emerged. Neutrophils exhibit anti-tumor responses concurrently with pro-tumor activities in tumor microenvironment. Neutrophils limit tumor growth through direct, antibody-dependent cytotoxic effects and activation of immune cells. On the other hand, cancer cells induce the systemic activation of neutrophil extracellular matrix traps, which lead to increased adhesion, destruction of basement membrane, invasion of cancer cells and metastasis^39^. The cytokines from neutrophil, including vascular endothelial growth factor, fibroblast growth factor 2, oncostatin M, matrix metalloproteinase 9^39–41^ and elastase^42^ are involved in chronic inflammation and cancer progression. In addition, neutrophil has been found to be related with suppression of cell-mediated immunity for cancer surveillance^43^. Rao et al. reported that elastase from neutrophils can also inhibit recruitment of T lymphocyte into the inflammation sites^42^. An investigation by Gabrilovich et al. revealed the T cell suppression mechanism whereby myeloid-derived suppressor cells overproduce the reactive oxygen species and arginase 1^44^. Also, there have been several explanations for the association between platelets and cancer progression. Platelets can interact with tumor cells and provide mechanical support on them^45^. Sabrkhany et al. confirmed that platelets can also promote cancer cell proliferation and metastasis by increasing angiogenesis and vessel permeability^46^. The study of Nieswandt et al. revealed that platelets also defend cancer cells from the host immune system by diminishing the cytotoxic activity of natural killer cells^47^.

Meanwhile, elevated NLR and PLR also result from relative lymphopenia. Possible mechanisms for lymphopenia and inferior survival outcome in cancer patients have been suggested. The lymphocyte is known to be a critical component of anticancer immunity in the form of adaptive immune response. A low lymphocyte count might underlie an insufficient host immune response^48^ due to destruction of lymphocytes by cancer cells^49^. Consequently, the risk of cancer development and progression might increase in immunocompromised, lymphopenia population^48^.

Given the retrospective nature of present study, there is a possibility of bias in patient selection and the results cannot be readily extrapolated to the general population. More data are needed to set the optimal cut-off values of SIMs that can be applied in clinical situations. In the future, a multicenter, prospective cohort study should be warranted for incorporating SIMs into a practical staging tool for OSCC. Another limitation is that our cohort included only patients who had undergone primary surgery-based treatment for OSCC. Patients who had received primary radiotherapy and/or chemotherapy were not included. Although there is no noticeable difference between the cut-off values according to the treatment modalities for OSCC, discreet approaches are required to interpret the findings of this study.

## Conclusion

A precise prediction of survival before curative surgery in OSCC is still challenging. In the framework of AJCC 8th edition, the present study demonstrated that elevated NLR or PLR can be another preoperative clue to identify the patients who are in risk of shorter survival and higher recurrence. When pathologic data was included in multivariate analysis, elevated NLR and pN were independent predictors for poor OS and DFS, and PNI for worse OS. Although SIM need to be further validated, NLR is suggested to be of value in predicting survival outcomes during preoperative and postoperative assessment.

## Supplementary information

Supplementary file1 (PDF 425 kb)
