# Supplementary material for: Prognostic value of systemic inflammatory markers for oral cancer patients based on the 8th edition of AJCC staging system
Source: Sci Rep. 2020 Jul 21;10:12111. doi: 10.1038/s41598-020-68991-3 (PMC7374730; doi:10.1038/s41598-020-68991-3)
Supplement: Supplementary file 1 — Supplementary file1 (PDF 425 kb) [file 41598_2020_68991_MOESM1_ESM.pdf]

Supplementary Information

Article in *Scientific Reports*

Prognostic value of systemic inflammatory markers for oral cancer patients based on the 8th edition of AJCC staging system

Sanghoon Lee, Dong Wook Kim, Sunmo Kwon, Hyung Jun Kim, In-Ho Cha, Woong Nam

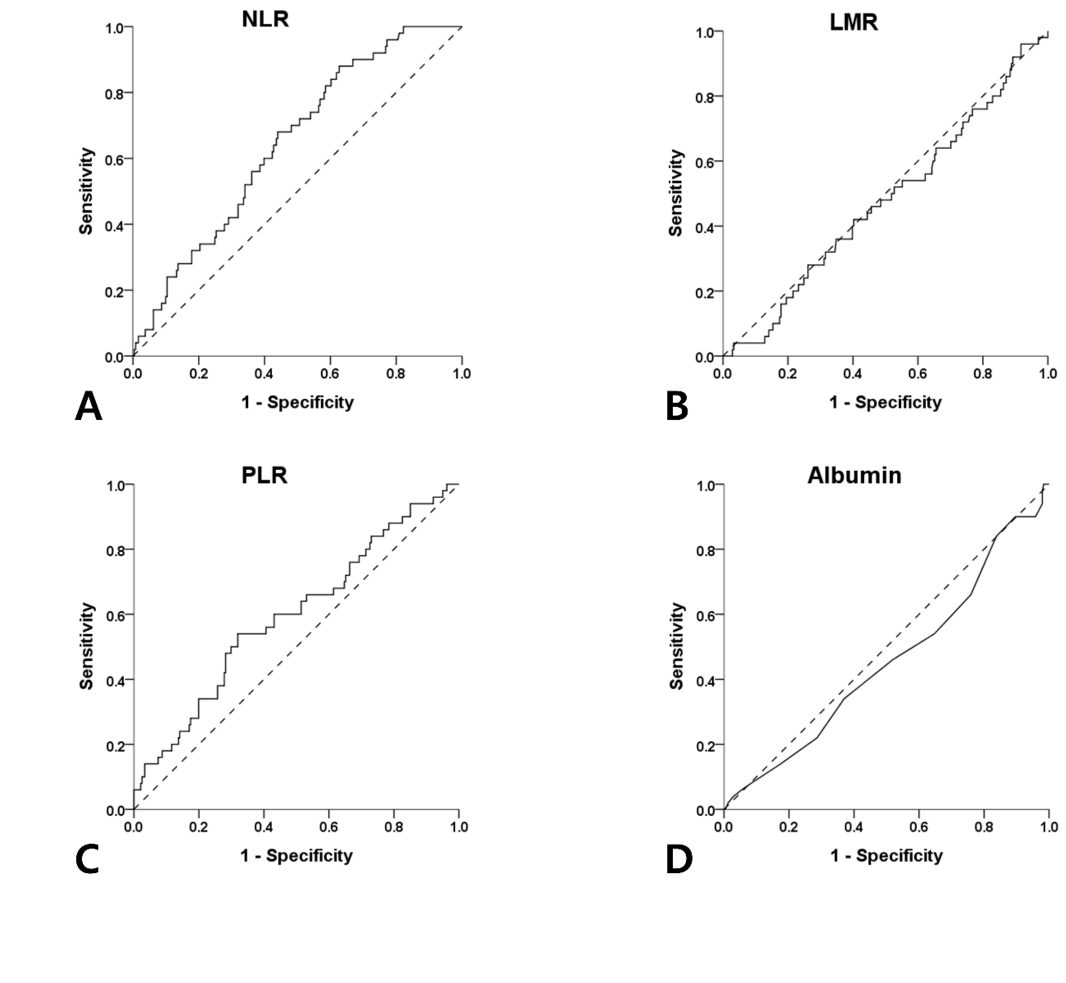

Supplementary Figure S1. Receiver operating characteristic curves for determining optimal cut-off value of the preoperative systemic inflammatory markers for overall survival: (A) neutrophil-lymphocyte ratio (AUC = 0.640, $P < 0.01$); (B) lymphocyte-monocyte ratio (AUC = 0.482, P = 0.689); (C) platelet-lymphocyte ratio (AUC = 0.595, P = 0.033); (D) Albumin (AUC = 0.454, P = 0.307)

Supplementary Table S1. Receiver operating characteristic curve analysis for overall survival

**Parameter**	**NLR**	**LMR**	**PLR**	**Albumin**
AUC	0.640	0.482	0.595	0.454
95% CI	0.56-0.72	0.40-0.57	0.51-0.68	0.37-0.54
Optimal sensitivity (%)	58.8	49.0	56.9	46.0
Optimal specificity (%)	60.3	51.7	59.5	51.9
Cut-off value at optimal sensitivity	2.23	4.65	135.14	4.35
p-value	0.002*	0.689	0.033*	0.307

Abbreviation: NLR, neutrophil-lymphocyte ratio; LMR, lymphocyte-monocyte ratio; PLR, platelet-lymphocyte ratio; AUC, area under the curve; CI, confidence interval

* Statistically significant

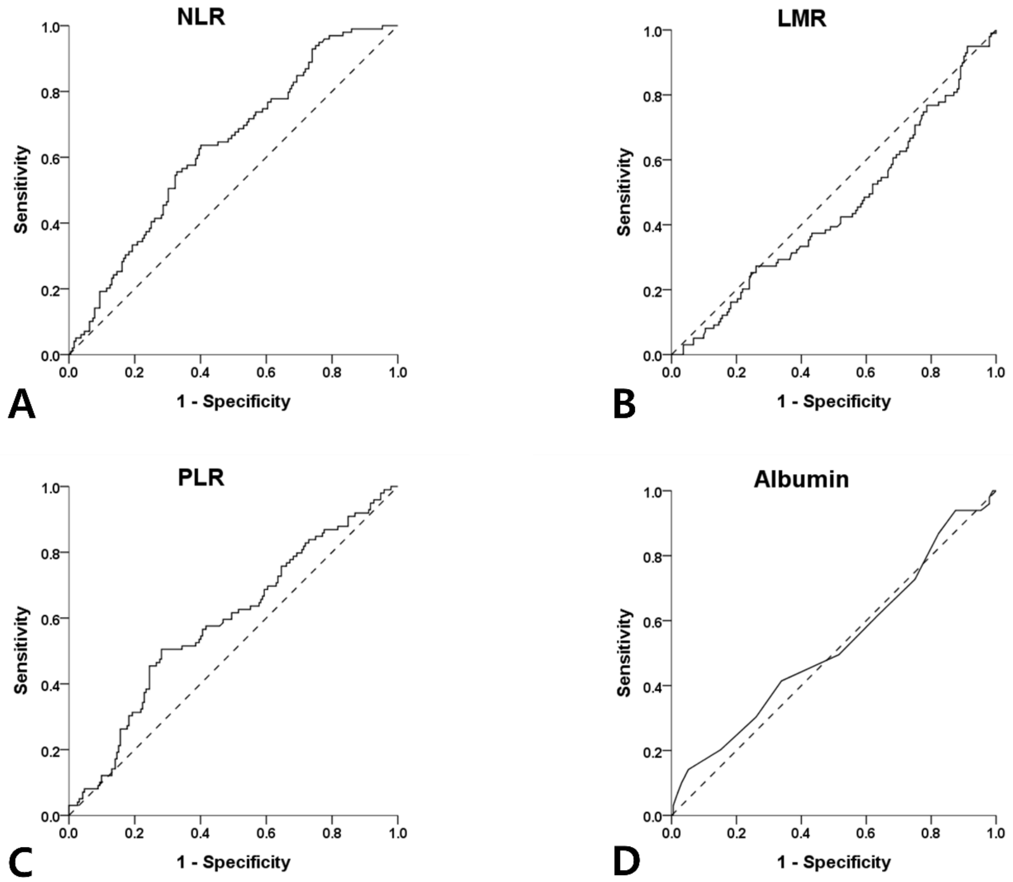

Supplementary Figure S2. Receiver operating characteristic curves for determining optimal cut-off value of the preoperative systemic inflammatory markers for disease-free survival: (A) neutrophil-lymphocyte ratio (AUC = 0.630, P ＜ 0.01); (B) lymphocyte-monocyte ratio (AUC = 0.450, P = 0.163); (C) platelet-lymphocyte ratio (AUC = 0.588, P = 0.014); (D) Albumin (AUC = 0.525, P = 0.484)

Supplementary Table S2. Receiver operating characteristic curve analysis for disease-free survival

**Parameter**	**NLR**	**LMR**	**PLR**	**Albumin**
AUC	0.630	0.450	0.588	0.525
95% CI	0.56-0.70	0.38-0.52	0.52-0.66	0.45-0.60
Optimal sensitivity (%)	62.0	44.0	58.0	57.6
Optimal specificity (%)	60.1	44.6	58.0	42.2
Cut-off value at optimal sensitivity	2.16	4.45	130.32	4.35
p-value	<0.001*	0.163	0.014*	0.484

Abbreviation: NLR, neutrophil-lymphocyte ratio; LMR, lymphocyte-monocyte ratio; PLR, platelet-lymphocyte ratio; AUC, area under the curve; CI, confidence interval

* Statistically significant
